# Phytocompound screening, antioxidant activity and molecular docking studies of pomegranate seed: a preventive approach for SARS-CoV-2 pathogenesis

**DOI:** 10.1038/s41598-023-43573-1

**Published:** 2023-10-10

**Authors:** Fauzia Ashfaq, Md. Abul Barkat, Tanvir Ahmad, Mohd. Zaheen Hassan, Rumana Ahmad, Harshita Barkat, Mohammad Idreesh Khan, Fahad Saad Alhodieb, Yahya I. Asiri, Sahabjada Siddiqui

**Affiliations:** 1 Clinical Nutrition Department, Applied Medical Sciences College, Jazan University, Jazan 82817, Saudi Arabia; 2 Department of Pharmaceutics, College of Pharmacy, University of Hafr Al-Batin, Al Jamiah, 39524 Hafr Al Batin, Saudi Arabia; 3 Department of Biotechnology, Era’s Lucknow Medical College and Hospital, Lucknow, 226003 India; 4 Department of Pharmaceutical Chemistry, College of Pharmacy, King Khalid University, Abha, Saudi Arabia; 5 Department of Biochemistry, Era’s Lucknow Medical College and Hospital, Lucknow, 226003 India; 6 Department of Clinical Nutrition, College of Applied Health Sciences in Ar Rass, Qassim University, Ar Rass 51921, Saudi Arabia; 7 Department of Pharmacology, College of Pharmacy, King Khalid University, Abha, Saudi Arabia

**Keywords:** Computational biology and bioinformatics, Drug discovery, Plant sciences

## Abstract

A global hazard to public health has been generated by the coronavirus infection 2019 (COVID-19), which is spreading quickly. Pomegranate is a strong source of antioxidants and has demonstrated a number of pharmacological characteristics. This work was aimed to analyze the phytochemicals present in ethanolic pomegranate seed extract (PSE) and their in vitro antioxidant potential and further in-silico evaluation for antiviral potential against crystal structure of two nucleocapsid proteins *i.e.,* N-terminal RNA binding domain (NRBD) and C-terminal Domain (CTD) of SARS-CoV-2. The bioactive components from ethanolic extract of PSE were assessed by gas chromatography-mass spectroscopy (GC–MS). Free radical scavenging activity of PSE was determined using DPPH dye. Molecular docking was executed through the Glide module of Maestro software. Lipinski’s 5 rule was applied for drug-likeness characteristics using cheminformatics Molinspiration software while OSIRIS Data Warrior V5.5.0 was used to predict possible toxicological characteristics of components. Thirty-two phytocomponents was detected in PSE by GC–MS technique. Free radical scavenging assay revealed the high antioxidant capacity of PSE. Docking analysis showed that twenty phytocomponents from PSE exhibited good binding affinity (Docking score ≥ − 1.0 kcal/mol) towards NRBD and CTD nucleocapsid protein. This result increases the possibility that the top 20 hits could prevent the spread of SARS-CoV-2 by concentrating on both nucleocapsid proteins. Moreover, molecular dynamics (MD) simulation using GROMACS was used to check their binding efficacy and internal dynamics of top complexes with the lowest docking scores. The metrics root mean square deviation (RMSD), root mean square fluctuation (RMSF), intermolecular hydrogen bonding (H-bonds) and radius of gyration (Rg) revealed that the lead phytochemicals form an energetically stable complex with the target protein. Majority of the phytoconstituents exhibited drug-likeness with non-tumorigenic properties. Thus, the PSE phytoconstituents could be useful source of drug or nutraceutical development in SARS-CoV-2 pathogenesis.

## Introduction

A coronavirus disease (COVID19) instigated by the SARS-CoV-2 is currently the subject of global attempts to protect people from it. COVID19 began at the end of 2019 from a non-human source in the Chinese province of Wuhan. According to the World Health Organization (WHO), COVID-19 is a global epidemic that is causing serious economic and health consequences in several countries. Globally, as of August 09, 2023, approx. 769,369,823 confirmed cases of COVID-19, including 6,954,336 deaths have been reported. A total of 13,492,264,486 vaccine doses have been administered on August 07, 2023 (WHO, 2023, https://covid19.who.int/). Even though the rising frequency of COVID-19 has been curbed by vaccination, there are increased death rates for a greater number of severe cases in geriatric patients. To improve outcomes in COVID-19 patients, there is currently no specific medication against SARS-CoV-2 that is available. The creation of powerful drugs may provide a long-term cure for COVID-19 and associated diseases^1^. In this sense, considering its potential benefits in terms of availability, cost, and usability, herbal medicine may be researched in the creation of powerful medications.

With the aid of numerous ways and methodologies, natural items might be demonstrated to be a safe and effective “natural therapy” for lung disorders treatment. Natural products have advantages due to their extensive scaffold variety and complicated structural composition, which may benefit with drug-disease interactions. Moreover, many herbal products have been proven a safe and effective “natural remedy” for viral and cancer disease treatment^1,2^. However, the selection of plant species is key point for the development of effective anti-SARS-CoV2 medicine to combat COVID-19. Numerous studies have looked into the possibility of using herbal medicine to treat and avoid the spread of COVID-19 by obstructing SARS-CoV-2 growth^3,4^. *Punica granatum*, also referred to as pomegranate, is a type of herbal plant that has demonstrated promising pharmacological properties. The entire fruit, including the seeds and peels, is rich in several classes of active ingredients, including fatty acids and organic acids, phenolics, flavonoids, steroids, tannins, anthocyanins, proanthocyanidins, alkaloids, terpenes and terpenoids, xanthonoids, lignans, saccharides, and vitamin C^5^. Along with medicinal and nutritional benefits, pomegranate entire fruits, peels, juice, and seeds are used to prepare beverages and jams^6^. Numerous therapeutic characteristics of pomegranate, including anti-inflammatory, antibacterial, anticarcinogenic, antioxidant, anti-inflammatory, and cardiovascular protection, have been demonstrated by various studies^7^. Among other parts of plant, pomegranate seeds are the rich source of antioxidants, which help to protect the human body against inflammation and free radical damage^8^. In a previous study, pomegranate seed oil significantly decreased the skin tumor incidence and multiplicity induced by 12-*O*-tetradecanoylphorbol 13-acetate (TPA) in CD1 mice^9^. Recent study has shown that pomegranate seed oil reduced the cell proliferation of esophageal cancer cell line- KYSE-30^10^.

Because of the importance of several classes of bioactive compounds in pomegranate seeds, this study was aimed to investigate the phytochemicals present in pomegranate seeds extract (PSE), var. Jalore seedless through GC–MS technique and their antioxidant activity using DPPH dye. To address the immediate need for COVID-19 treatment, this study was extended to find the binding interaction of the identified phytochemical through computational molecular docking analysis against two targeted nucleocapsid proteins viz*.* N-terminal RNA binding domain (NRBD; PDB ID: 6M3M) and C-terminal Dimerization Domain (CTD; PDB ID: 6WJI) from SARS-CoV-2. The most conserved structural protein that binds the viral RNA genome for viral replication and transcription is the nucleocapsid protein. Nucleocapsid protein structure comprises an NRBD, a CTD and a naturally chaotic center rich in Arg/Ser linker^11^. Thus, focusing on these particular targets may establish a reasonable strategy for drug or nutraceutical development.

## Materials and methods

### Collection of *Punica granatum* and extract preparation

In month of February, fresh fruits of cultivated *Punica granatum* plant**,** var. Jalore seedless were collected from Jodhpur, Rajasthan, India. Pomegranate fruits were identified, and submitted at Pharmacognosy Department, Integral University, Lucknow (File No. IU/PHAR/HRB/14/08). Pomegranate seeds were separated, rinsed with ddH_2_O, dried in the shade, and then ground into a powder using a Bajaj grinder. A coarse powder was steeped in 95 percent ethanol for three days at 25 °C in order to remove any soluble components. The filtrate was separated from the supernatant using a Whatman No. 1 filter membrane, and the ethanol was evaporated using a Rotatory evaporator. To obtain the semi-dried form of pomegranate seed extract, the resultant semi-solid paste was further evaporated on a water bath. All procedures were followed as per guidelines: https://www.biomedcentral.com/getpublished/editorial-policies#research+involving+plants.

### GC–MS based analysis

Gas chromatography–mass spectroscopy is an ideal technique for phytoconstituents characterization viz*.* to predict their name, structure, formula and retention time. As per previous study, RTX-5 MS capillary column (Restek) was used to chemically characterize the 95% ethanolic PSE with the help of GC-MS-QP2010 Plus system (Shimadzu, Japan)^12^. GC separates the various constituents into their individual components with their variable retention times and mass spectrophotometer identifies the components. A chromatogram of relative abundance against retention time was produced by the software, coupled to the mass spectrophotometer. Comparisons between the mass spectra of known and unknown components were conducted by NIST08 and WILEY8 chemistry libraries. It was possible to determine the components' names, molecular weights, m/z values, and structures.

### Free radical scavenging activity of PSE

The free radical scavenging capacity of PSE was assessed by 2, 2-diphenyl-2-picryl-hydrazyl (DPPH) according to a previously described procedure^13^. Briefly, 2 mL of 0.1 mM DPPH solution made in methanol was added to various concentrations of PSE prepared in 1% DMSO and adjusted to 3 mL using DPPH solution. The mixtures were shaken and allowed for 30 min at RT. Absorbance was determined at 517 nm with the help of a UV–Vis double beam spectrophotometer (Systronics, 2203). Low absorbance value shows high free radical scavenging activity. DPPH scavenging capacity (% inhibition) = [(Ac−As)/Ac)*100]; Ac, absorbance of the control reaction; As, absorbance of PSE.

### Computational study

#### Preparation of phytoconstituents for molecular docking

Thirty-two phytomolecules from pomegranate seed as well as ivermectin and hydroxychloroquine, two standard chemicals, were downloaded into SDF format from the PubChem database. Through the LigPrep module of Schrodinger Maestro v2020-2, these were turned into three-dimensional configurations by the addition of hydrogen atoms, tautomer creation, establishing ionization states, charged groups neutralization, geometry optimization and structure filtration using OPLS 2005. At pH 7.0 +/− 2.0, ligands were ionized using the Epik module, which produced tautomers, desalted them, and carried out a single low energy ring confirmation for each ligand. Table 1 displays the 2-D structure, PubChem CID, molecular formula, percentage area, and retention time of detected phytoconstituents.

**Table 1:** The identified phytoconstituents and molecular weight (MW), molecular formula (MF), retention time (RT), %Area, and nature of compounds from PSE through GC–MS technique.

S. No.	Compounds Name	Structure	PubChem ID	MF	MW	Area %	RT (min)	Fragments (m/z)	Nature
1.	Imidazolidine-2,4,5-trione		67126	C3H2N2O3	114	0.47	3.166	43,114	Pyranone
2.	2,5-Dimethyl-4-hydroxy-3(2H)-furanone		19309	C6H8O3	128	1.32	3.270	43,57,128	Pyranone
3.	Cyclopentane, 1-acetyl-1,2-epoxy-		537123	C7H10O2	126	11.36	3.631	43,83,126	Cyclopentane
4.	4H-Pyran-4-one, 2,3-dihydro-3,5-dihydroxy-6-methyl		119838	C6H8O4	144	11.20	4.582	43,144,101	Pyranone
5.	2(3H)-Furanone, dihydro-4-hydroxy-		95652	C4H6O3	102	2.03	4.917	44,74,102	Pyranone
6.	2-Butanone, 4-hydroxy-3-methyl		18829	C5H10O2	102	4.03	5.259	43,61,31	Alcoholic ketone
7.	2-Furancarboxaldehyde, 5-(hydroxymethyl)		237332	C6H6O3	126	22.78	5.880	97,41,126	Furan (Alcohol)
8.	2,3-dihydroxypropyl acetate		33510	C5H10O4	134	4.52	6.127	43,103	Alcohol
9.	Heptanoic acid, 6-oxo-		98810	C7H12O3	144	2.86	7.010	43,58,126	Fatty acid ketone
10.	1-O-hexyl-d-glucitol		537891	C12H26O6	266	0.88	7.832	43,73,61	Alcohol
11.	5H-Imidazole-4-carboxylic acid, 5-amino-, ethyl ester		6424292	C6H9N3O2	155	3.86	8.277	155,109,127	Aromatic heterocycle (alkaloid)
12.	Ethyl 5-oxo-2-pyrrolidinecarboxylate		2724446	C7H11NO3	157	0.30	8.942	84,41,157	Amine
13.	Phenol, 2,4-bis(1,1-dimethylethyl)		7311	C14H22O	206	0.63	9.712	191,57,206	Polyphenol
14.	Ethyl .alpha.-d-glucopyranoside		11127487	C26H54	366	1.25	11.858	60,42,73	Glucopyranoside
15.	3-Deoxy-d-mannoic lactone		541561	C6H10O5	162	5.74	12.292	44,57,102	Lactone derivative
16.	Tetradecanoic acid		11005	C14H28O2	228	3.10	12.623	60,73,129	Fatty acid
17.	7,9-Di-tert-butyl-1-oxaspiro(4,5)deca-6,9-diene-2,8-dione		545303	C17H24O3	276	0.26	14.350	57,205,55	Ketone
18.	n-Hexadecanoic acid		985	C16H32O2	256	6.55	14.682	73,60,43	Saturated fatty acid
19.	Hexadecanoic acid, ethyl ester		12366	C18H36O2	284	0.74	14.949	88,101,41	Fatty acid ester
20.	Oleic acid		445639	C18H34O2	282	5.06	16.361	55,41,69	Fatty acid
21.	Octadecanoic acid		5281	C18H36O2	284	3.94	16.547	43,73,60	Stearic acid
22.	Octadecanal		12533	C18H36O	268	0.23	19.960	43,57,82	Aldehyde
23.	gamma-Tocopherol		92729	C28H48O2	416	0.15	27.337	151,416,191	Sterol
24.	3.alpha.,12.beta.-Dihydroxy-bisnor-5,7-cholenic acid		620404	C22H32O4	360	0.10	27.442	255,342,273	Steroid
25.	Cholesta-4,6-dien-3-ol, (3.beta.)		14795191	C27H44O	384	0.10	27.550	43,135,366	Steroid
26.	Stigmast-5-en-3-ol, oleate		20831071	C47H82O2	678	0.25	27.716	147,396,382,367	Steroid
27.	DL-.alpha.-tocopherol		2116	C29H50O2	430	0.13	27.958	165,430,43	Vitamin E
28.	Hexadecanoic acid, tetradecyl ester		78294	C30H60O2	452	0.55	28.531	57,257,43	Fatty acid ester
29.	Ergost-5-en-3-ol, (3.beta.,24R)		6428659	C28H48O	400	0.60	28.751	107,95,400,382,367	Phytosterol
30.	Stigmasta-5,22-dien-3-ol		53870683	C29H48O	412	0.26	29.002	55,83,412,351	Phytosterol
31.	Stigmast-5-en-3-ol, (3.beta.)		6432744	C29H50O	414	2.58	29.484	43,81,396,381,414	Phytosterol
32.	Fucosterol		5281328	C29H48O	412	0.24	29.639	81,57,43,396,381,414	Phytosterol

#### Preparation of crystal proteins

The NRBD; PDB ID: 6M3M, an A1 protein monomer, and the CTD; PDB ID: 6WJI, an A2 protein homo 2-mer, both from SARS-CoV-2, were downloaded from protein data bank in PDB format. Without any mutation, the NRBD and CTD had X-ray diffraction resolution values of 2.70 and 2.05, respectively. The side-chain hydroxyl groups Gln, His and Asn states were optimized using the OPLS 2005 force field by establishing bond ordering, adding missing hydrogen atoms and disulfide links, removing water molecules within 5 of the heteroatoms, and replacing missing disulfide bonds.

#### Receptor grid setup

Receptor grid generation was utilized to know the interactions between different ligands and receptor proteins. The docked ligand’s position was constrained to the workspace ligand’s reduced size, the centroid of the docked pose, and the surrounding box. The grid generation did not include the initial bounded ligand.

#### Standard precision (SP) for Glide Docking

Following the preparation of the ligands and proteins, the glide SP flexible ligand mode of Schrödinger Maestro Release 2020-2 was used to dock the ligands with the proteins in 10 poses each^14^. The OPLS3e force field was used in the glide SP flexible docking analysis. Poses with least energy were selected, and the Glide score was used to determine the final score. The lowest glide score and the best-docked orientation for each ligand were documented. The docking scores of phytocomponents, which represent the binding energies, were then used to organize them.

#### Interpreting and visualizing docking results

Data in the form of glide energy and glide score were produced following the glide docking study with SP mode. The top-ranking compounds were sorted based on Glide score to identify the appropriate binding interaction complex. The 2D interactions with the best poses, such as hydrogen bond contacts, wander Waal interactions, and hydrophobic interactions, were also displayed and analysed using SP visualizer as a ligand interaction tool. With the aid of the software LigPlot + v.2.2, the binding pattern of the best docking results was further examined.

#### Molecular dynamics simulation

Following the docking studies, lead compounds obtained from docking analysis *i.e.* 1. 3.alpha.,12.beta.-Dihydroxy-bisnor-5,7-cholenic acid with NRBD protein and 2. ethyl 5-oxo-2-pyrrolidinecarboxylate with CTD domain of SARS-CoV-2 were used for MD simulation study to check their binding efficacy and internal dynamics of both complex^15^. The GROMACS (Version 2023.2) was utilized to perform MD simulation. CHARMM27 force field and transferable intermolecular potential 3P (tip3p) water model was applied to conduct MD simulation of both complexes^16^. SwissParam was used to generate force field parameters for the phytochemicals. Na and Cl ions were used to balance complex charges. Simulations were run at a pressure of 1 bar and a temperature of 300 K. The steepest descent algorithm was used to reduce each system in 5000 steps. The Particle-Mesh-Ewald (PME) summation was used to calculate electrostatic interactions^17^. By running 1000 kJ/mol nm^2^ position restraint simulations for 100 ns in the NVT and NPT ensembles, the systems were brought to equilibrium. A 100 ns no restraint production run was simulated using equilibrated systems. Root mean square deviation, root mean square fluctuations, radius of gyration, and hydrogen bond occupancy are some of the post-MD analyses that were carried out. Utilizing the XMGrace program, the entire plot was created^18^.

### Lipinski’s 5 rule for drug-likeness

Lipinski’s rule of 5 was used to assess the drug-likeness of the top twenty active components of PSE^19^. These estimates had included hydrogen bond donors number (NOHNH) ≤ 5, topological polar surface area (TPSA) (≤ 140 Å^2^), hydrogen bond acceptor number (NON) ≤ 10, rotatable bond number ≤ 10, MW ≤ 500, and logP ≤ 5. Twenty active ingredients were assessed for their drug-likeness using the cheminformatics program Molinspiration (https://www.molinspiration.com/).

### Potential toxicity study

When developing pharmaceutically active compounds, the preliminary knowledge of the physico-chemical and potential toxicological molecular characteristics of constituents needs to be optimized. Predicting various phytocomponent features at an early stage is essential for lead research and development. Software application OSIRIS Data Warrior V5.2.1 was used to forecast physico-chemical and toxicological molecular features like mutagenic, drug-likeness, tumorigenic, reproductive, and irritating effects^20^.

## Results and discussion

### GC–MS analysis of pomegranate seed extract

**Figure 1:**
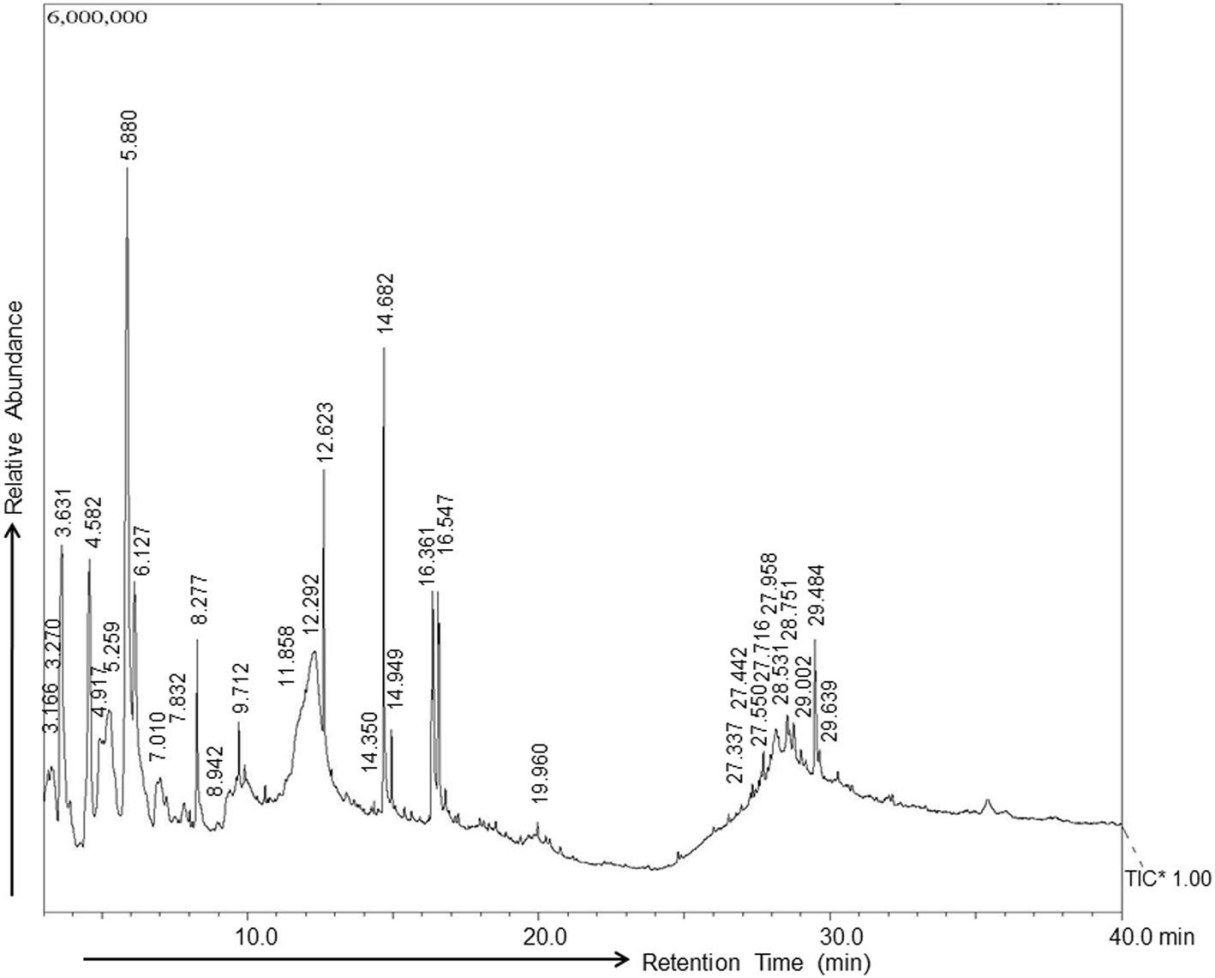
Total ion chromatogram, showing the intensities of all mass spectral peaks belonging to the same scan of the identified compounds from pomegranate seed extract (95% ethanolic). Y-axis is total ion current; X-axis is retention time.

Traditional herbal medicines provide vital role in health sectors by improving various acute and chronic conditions without or minimal toxic effect. It is essential to identify the chemical nature and their medicinal properties of phytocomponents present in traditional herbs. This method might be able to prove experimentally the traditional use of medicinal plants. Small group of acids, hydroxyl acids, amino acids, fatty acids, sugars, alcohols, sterols, catecholamines, toxins and drugs are among the small molecular metabolites that are ideal for GC–MS-based metabolomics. These compounds are frequently chemically modified to make them volatile enough for gas chromatography^21^. While MS uses a plot of the ion signal as a function of the mass-to-charge ratio to determine the elemental profile, chemical makeup, or structural characteristics of chemical compounds, GC is utilized to distinguish between volatile and thermally stable alternatives in a sample. Natural occurring volatile components are investigated using by GC–MS analysis and those having semi- or non-volatile metabolites can be induced into volatile through derivatization and then can be investigated using GC–MS. Using effective derivatization techniques involving silylation, alkylation or acylation reactions, a wide range of semi- and non-volatile metabolites, including sugars, sugar alcohols, sugar phosphates, organic acids, lipids, flavonoids, peptides, amino acids, amides, amines, long-chain alcohols, and alkaloids can also be studied^22,23^. In this GC–MS analysis, silylation processes was employed to detect and identify semi-volatile and non-volatile components of pomegranate seed extract. The chromatogram of GC–MS analysis showed thirty-two peaks representing the presence of thirty-two components in pomegranate seed alcoholic extract. Figure 1 represents the total ion current (TIC) chromatogram of the pomegranate seed extract. The identified phytochemicals from alcoholic extract of native pomegranate seed, molecular weight, percent peak area and their retention time described in Table 1. The results revealed that the major constituents were found to be 2-Furancarboxaldehyde, 5-(hydroxymethyl) (22.78%), Cyclopentane, 1-acetyl-1,2-epoxy-(11.36%), 4H-Pyran-4-one, 2,3-dihydro-3,5-dihydroxy-6-methyl (11.2%), *n*-Hexadecanoic acid (6.55%), 3-Deoxy-d-mannoic lactone (5.74%), Oleic acid (5.06%), 2,3-dihydroxypropyl acetate (4.52%), 2-Butanone, 4-hydroxy-3-methyl (4.03%), Octadecanoic acid (3.94%), 5H-Imidazole-4-carboxylic acid, 5-amino-, ethyl ester (3.86%), Tetradecanoic acid (3.1%), Heptanoic acid, 6-oxo-(2.86%), Stigmast-5-en-3-ol, (3.beta.) (2.58%) and 2(3H)-Furanone, dihydro-4-hydroxy (2.3%). The minor phytocomponents were 2,5-Dimethyl-4-hydroxy-3(2H)-furanone (1.32%), ethyl .alpha.-d-glucopyranoside (1.25%), 1-*O*-hexyl-d-glucitol (0.88%), Hexadecanoic acid, ethyl ester (0.74%), Phenol, 2,4-bis(1,1-dimethylethyl) (0.63%), Ergost-5-en-3-ol, (3.beta.,24R) (0.60%), Hexadecanoic acid, tetradecyl ester (0.55%), Imidazolidine-2,4,5-trione (0.47%), Ethyl 5-oxo-2-pyrrolidinecarboxylate (0.30%), Stigmasta-5,22-dien-3-ol and 7,9-Di-tert-butyl-1-oxaspiro(4,5)deca-6,9-diene-2,8-dione (0.26%), Stigmast-5-en-3-ol, oleate (0.25%), Fucosterol (0.24%) and Octadecanal (0.23%). All other components were identified in trace amount. In an earlier study, hexane: ethanoic (3:1) extracts of pomegranate seed oil was analyzed by GC–MS, which identified 23 compounds, the majority of which belonged to fatty amides and fatty acids^24^. Another study has shown that pomegranate seed oils include polyunsaturated long-chain fatty acids as one of the primary components^25^. Approx. thirty-eight phytochemicals were found in the Pomegranate fruit extract, with the majority containing alkaloids, fatty amides, and indazole derivatives^26^. Fatty acids, terpenes, heterocyclic compounds, and flavonoids types of compounds were found in the pomegranate leaf extract^27^. In a similar study, GC–MS analysis revealed the presence of twenty-three chemicals in pomegranate peel extract, with predominantly secondary alcohols, organosulfur compounds, and fatty acid derivatives^28^. In the current study, GC–MS analysis identified thirty two phytocompounds, with the majority containing pyranones, fatty acids, alkaloids and phytosterols (Table 1). Numerous studies have demonstrated the antiviral effects of plant metabolites such pyranone, fatty acid ketone, alkaloid, polyphenol, phytosterol and fatty acid ester against a variety of viruses^28–31^. Based on these studies, the reported phytocomponents in PSE were further assessed for their potential as virtual antiviral agents utilizing Glide Docking in SP mode with Schrödinger Maestro Release 2020-2.

### Antioxidant activity of PSE

**Figure 2:**
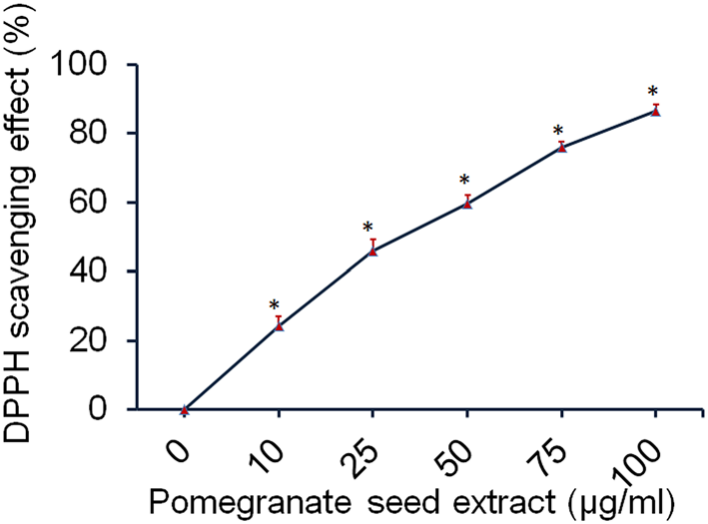
Antioxidant activity of ethanolic extract of PSE using DPPH free radical scavenging method. Values are shown as mean ± SD of three independent experiments. *p < 0.05 as compared to control.

DPPH radical scavenging activity of PSE is depicted as percent DPPH scavenging effects in Fig. 2. Results showed that the percent DPPH scavenging activity was found to be 24.3, 45.9, 59.5, 75.9 and 86.5% at 10, 25, 50, 75 and 100 µg/mL of PSE. This study suggested that free radical scavenging activity of DPPH radical was increased in a dose-dependent manner. The antioxidant capacity might be due to the high content of phenolic and flavonoid components in the ethanolic extract of PSE. Pomegranate seeds are a byproduct in the production of pomegranate juice. Pomegranate seeds possess potent antioxidants, and anti-inflammatory chemicals such as vitamin E, sterols, and phenols, plethora of fatty acids and natural estrogens^32^.

### Docking analysis of PSE phytocomponents

SP Glide docking of Schrödinger maestro v2020-2 was used to analyze the binding interactions of thirty-two phytocomponents from PSE against NRBD and CTD of nucleocapsid protein from SARS-CoV-2. The details of all Glide E energy, Gibbs binding free energy and docking scores of identified constituents with their respective NRBD and CTD proteins are mentioned in Tables 2 and 3. As shown in Table 2, twenty one phytocomponents (compounds’ serial numbers 1–11, 13–15 and 17–23) and standard drug hydroxychloroquine displayed good binding affinity (Docking score > − 1.00 kcal/mol) towards the NRBD nucleocapsid protein of SARS-CoV-2. Whereas, compounds’ serial numbers 12 and 16 displayed mild binding affinity (Glide score < − 1.00 kcal/mol). Two compounds exhibited positive docking score, while remaining compounds and ivermectin did not exhibit any binding interaction suggesting little/no affinity towards proteins (Table S1). More negative values of the Docking score which imitate binding free energy indicating stronger binding between ligand–protein interactions. The highest docking score was displayed by 3.alpha.,12.beta.-dihydroxy-bisnor-5,7-cholenic acid with NRBD nucleocapsid protein (Docking score = − 7.697). In case of CTD nucleocapsid protein (Table 3), 19 phytocomponents that showed a negative glide score, eighteen components displayed more than − 1.0 kcal/mol of Docking score. Compound ethyl 5-oxo-2-pyrrolidinecarboxylate displayed the best docking score (Docking score = − 6.705 kcal/mol) towards CTD nucleocapsid protein. Compounds’ serial number 7 displayed mild binding affinity (Glide score < − 1.00 kcal/mol). While remaining compounds and ivermectin drug did not display any binding interaction and /or positive Docking score with CTD protein (Table S2). Alternatively, the standard compound hydroxychloroquine displayed a negative Docking score (− 3.831 kcal/mol). This study confirms the good binding affinity of hydroxychloroquine against both NRBD and CTD nucleocapsid protein, representing efficient inhibitor of SARS-CoV-2 in comparison to ivermectin.

**Table 2:** Docking score, Glide E model, Gibbs binding free energy, Glide energy, interacting amino acids and 2-D interaction diagram of the docked ligand–protein complex of 23 PSE components and reference drug with crystal structure of NRBD; PDB ID: 6M3M of SARS-CoV-2 nucleocapsid protein using glide SP module of Schrödinger.

S. No.	Ligands	PubChem CID	Docking score (kcal/mol)	Glide E model (kcal/mol)	Glide energy (kcal/mol)	Gibbs binding free energy (kcal/mol)	Interacted amino acid	2-D structure of ligand–protein interaction
1.	Imidazolidine-2,4,5-trione	67126	− 5.954	− 34.75	− 25.646	− 13.519	Ile D:158, Ala D:156, Asn D:155, Gly A:117, Thr A:149, Gly A:148, Trp D:53	
2.	2,5-Dimethyl-4-hydroxy-3(2H)-furanone	19309	− 5.187	− 25.847	− 17.744	0.07	Ile D:158, Phe D:54, Trp D:53, Ser D:79, Asn D:76, Tyr D:113, Asn B:127, Ile D:147, Asn A:49	
3.	Cyclopentane, 1-acetyl-1,2-epoxy-	537123	− 5.233	− 25.483	− 20.161	15.224	Trp D:53, Ile D:158, Asn D:76, Asn A:49, Asn D:78, Ser D:79, Ile D:147, Hie D:146, Gly B:125, Ala B:126, Asn B:127	
4.	2(3H)-Furanone, dihydro-4-hydroxy-	95652	− 5.371	− 25.844	− 19.727	− 2.291	Val D:159, Ile D:158, Ala D:157, Ala D:156, Asp A:145, Hie A:146, Ile A:147, Gly A:148, Thr A:149	
5.	2,3-dihydroxypropyl acetate	33510	− 3.515	− 27.649	− 24.095	− 1.455	Arg D:150, Trp D:53, Val D:159, Ile D:158, Ala D:157, Ala D:156, Asn D:155, Asn D:154, Ala A:120, Gly A:117, Thr A:116, Thr A:50, Asp A:145, Gly A:148, Thr A:149	
6.	Heptanoic acid, 6-oxo-	98810	− 3.996	− 28.016	− 22.57	3.399	Trp D:53, Asn B:127, Asn D:78, Asn D:76, Thr A:149, Thr A:50, Asn D:155, Ala D:156, Ile D:158, Gly A:117, Thr A:116	
7.	1-*O*-hexyl-d-glucitol	537891	− 2.905	− 44.839	− 41.047	8.658	Ser D:79, Asn D:78, Thr D:77, Asn D:76, Ile D:158, Tyr D:113, Phe D:54, Trp D:53, Thr A:149, Asn A:151, Thr A:50, Asn A:49, Ile D:147, Asn B:127	
8.	5H-Imidazole-4-carboxylic acid, 5-amino-, ethyl ester	6424292	− 4.439	− 28.829	− 22.463	4.205	Asn D:76, Asn D:78, Ser D:79, Asp D:83, Ile D:147, Hie D:146, Asn B:127, Ala B:126, Gly B:125, Phe D:54, Trp D:53, Tyr D:113, Ile D:158	
9.	Ethyl 5-oxo-2-pyrrolidinecarboxylate	2724446	− 6.087	− 40.19	− 29.583	1.858	Gly A:148, Thr A:149, Arg A:150, Asn A:151, Asn A:49, Thr A:50, Ala A:51, Thr D:77, Asn D:78, Trp D:53, Asn D:155, Ala D:156, Ile D:158, Gly A:117, Thr A:116	
10.	Ethyl .alpha.-d-glucopyranoside	11127487	− 5.458	− 39.234	− 30.165	12.598	Ile D:158, Asn D:155, Asn A:151, Thr A:149, Thr A:50, Asn A:49, Asn B:127, Ile D:147, Trp D:53, Asn D:76, Thr D:77, Asn D:78	
11.	3-Deoxy-d-mannoic lactone	541561	− 5.187	− 34.86	− 26.993	10.74	Ile D:158, Asn D:76, Thr D:77, Asn D:78, Trp A:53, Ala A:51, Thr A:50, Asn A:49, Thr A:149, Arg A:150, Asn A:151	
12.	Tetradecanoic acid	11005	− 0.112	− 26.068	− 29.047	7.846	Ile D:158, Ala D:156, Asn D:155, Thr A:116, Gly A:117, Ala A:120, Gly A:148, Thr A:149, Thr A:50, Pro B:123, Tyr B:124, Gly B:125, Ala B:126, Asn B:127, His D:146, Ile D:147, Trp D:53, Ser D:79, Asn D:78, Asn D:76	
13.	7,9-Di-tert-butyl-1-oxaspiro(4,5)deca-6,9-diene-2,8-dione	545303	− 6.677	− 48.576	− 35.578	22.094	Arg D:150, Val D:159, Ile D:158, Ala D:157, Ala D:156, Asn D:155, Trp D:53, Asp A:145, Hie A:146, Ile A:147, Gly A:148, Thr A:149, Arg A:150, Asn A:151, Gly A:117, Thr A:116, Asn D:78, Thr D:77, Asn D:76, Trp A:53, Ala A:51, Thr A:50, Asn A:49	
14.	Hexadecanoic acid, ethyl ester	12366	− 1.109	− 36.948	− 38.31	10.31	Val D:159, Ile D:158, Ala D:157, Ala D:156, Asn D:155, Asp A:145, Gly A:148, Thr A:149, Thr A:50, Ile D:147, His D:146, Asn B:127, Ala B:126, Gly B:125, Tyr B:124, Pro D:123, Asn D:76, Asn D:78, Ser D:79, Trp D:53	
15.	Oleic acid	445639	− 1.597	− 36.912	− 34.861	2.678	Ile D:158, Thr A:149, Ala D:156, Asn D:155, Ala A:120, Gly A:117, Thr A:116, Trp D:53, Asn D:76, Asn D:78, Ser D:79, Ser D:80, Asp D:83, Asn A:49, Thr A:50, Asn B:127, Ala B:126, Gly B:125, Tyr B:124, Pro B:123, Ile D:147, Hie D:146	
16.	Octadecanoic acid	5281	− 0.623	− 37.375	− 38.01	4.768	Asp D:83, Ser D:80, Asn D:78, Asn D:76, Thr A:50, Gly A:148, Thr A:149, Gly A:117, Asn D:154, Asn D:155, Ala D:156, Ile D:158, Ile D: 147, Hie D:146, Trp D:53, Asn B:127, Ala B:126, Gly B:125, Tyr B:124, Pro B:123	
17.	gamma-Tocopherol	92729	− 6.426	− 56.441	− 42.785	27.748	Val D:159, Ile D:158, Ala D:157, Ala D:156, Asn D:155, Asp D:83, Asn A:151, Thr A:149, Gly A:148, Ile A:147, Hie A:146, Asp A:145, Pro B:123, Tyr B:124, Gly B:125, Ala B:126, Asn B:127, Hie D:146, Ile D:147, Asn A:49, Thr A:50, Asn D:76, Thr D:77, Asn D:78, Ser D:79, Trp D:53, Tyr D:113	
18.	3.alpha.,12.beta.-Dihydroxy-bisnor-5,7-cholenic acid	620404	− 7.697	− 53.452	− 36.605	34.68	Gln D:161, Val D:159, Ile D:158, Ala D:157, Ala D:156, Asn D:155, Asp A:145, Hie A:146, Ile A:147, Gly A:148, Thr A:149, Arg A:150, Ile D:75, Asn D:76, Thr D:77, Asn D:78, Trp A:53, Thr A:50, Phe D:54, Trp D:53	
19.	DL-.alpha.-tocopherol	2116	− 5.959	− 56.413	− 43.513	28.148	Ile D:158, Ala D:157, Ala D:156, Asn D:155, Thr A:50, Asn A:49, Asn B:127, Ala B:126, Gly B:125, Asp A:145, Ile A:147, Gly A:148, Thr A:149, Asn A:151, Ala A:153, Trp D:53, Tyr D:113, Asn D:76, Thr D:77, Asn D:78, Ser D:79, Hie D:146, Ile D:147	
20.	Hexadecanoic acid, tetradecyl ester	78294	− 5.27	− 57.77	− 45.882	14.339	Asn A:76, Asn A:78, Arg A:150, Thr A:149, Gly A:148, Ile A:147, Hie A:146, Asp A:145, Gln D: 161, Val D:159, Ile D:158, Ala D:156, Asn D:155, Gly A:112, Tyr D:113, Ile D:147, Phe D:54, Trp D:53, Ser D:79, Asn D:78, Asn D:76, Asn D:127, Trp A:53, Thr A:50, Asn A:49, Ile A:158, Ala A:157, Ala A:156, Asn A:155	
21.	Ergost-5-en-3-ol, (3.beta.,24R)	6428659	− 5.837	− 48.008	− 38.24	52.243	Asp A:145, Hie A:146, Ile A:147, Asn A:155, Gly A:148, Thr A:149, Arg A:150, Asn A:151, Trp A:53, Thr A:50, Asn A:49, Asn D:76, Thr D:77, Asn D:78, Ser D:79, Asn B:127, Ile D:158, Asn D:155, Le D:147, Phe D:54, Trp D:53, Tyr D:113	
22.	Stigmasta-5,22-dien-3-ol	53870683	− 5.454	− 43.471	− 35.272	42.124	Gln D:161, Val D:159, Ile D:158, Ala D:157, Ala D:156, Asn D:155, Asp A:145, Hie A:146, Ile A:147, Gly A:148, Thr A:149, Asn A:151, Asn B:127, Gly B:125, Trp A:53, Thr A:50, Asn A:49, Trp D:53, Ile D:147, Asn D:78, Thr D:77, Asn D:76	
23.	Fucosterol	5281328	− 4.123	− 37.883	− 30.917	40.225	Gln D:161, Val D:159, Ile D:158, Ala D:156, Asn D:155, Asp A:145, Hie A:146, Ile A:147, Gly A:148, Thr A:149, Arg A:150, Asn A:151, Trp D:53, Trp A:53, Thr A:50, Asn A:49, Asn A:76, Thr D:77, Asn D:78	
24.	Hydroxychloroquine	3652	− 6.433	− 58.075	− 43.473	41.643	Tyr D:113, Trp D:53, Thr A:50, Asn A:49, Gly A:140, Thr A:149, Ile D:158, Ala D:156, Asn D:155, Arg D:150, Ile D:147, Hie D:146, Asn B:127, Ala B:126, Gly B:125, Tyr B:124, Asn D:76, Asn D:78, Ser D:79, Ser D:80, Asp D:83	

Note: 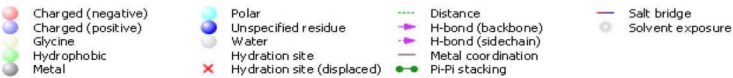

**Table 3:** Docking score, Glide E model, Gibbs binding free energy, Glide energy, interacting amino acids and 2-D interaction diagram of the docked ligand–protein complex of nineteen PSE phytocomponents and standard drug with CTD; PDB ID: 6WJI of SARS-CoV-2 nucleocapsid protein using glide SP module of Schrödinger.

S. No.	Ligands	PubChem CID	Docking score (kcal/mol)	Glide E model (kcal/mol)	Glide energy (kcal/mol)	Gibbs binding free energy (kcal/mol)	Interacted amino acid	2-D structure of ligand–protein interaction
1.	Imidazolidine-2,4,5-trione	67126	− 4.872	− 28.427	− 21.172	− 13.518969	Asp F:340, Leu F:339, Lys F:338, Ile F:337, Phe F:307, Gln E: 260, Arg E:259, Pro E:258	
2.	2,5-Dimethyl-4-hydroxy-3(2H)-furanone	19309	− 4.473	− 25.183	− 15.958	0.070381	Asp F:343, asp F:340, leu F:339, lys F;338, lys E:261, gln E:260, pro E:258, phe F:307, gln F:306, gln F:349	
3.	Cyclopentane, 1-acetyl-1,2-epoxy-	537123	− 4.671	− 25.053	− 20.286	15.2242	Lys F:338, leu F:339, asp F:340, asp F:343, gln F:349, gln F:306, phe F:307, gln E:260, arg E: 259, pro E:258	
4.	2(3H)-Furanone, dihydro-4-hydroxy-	95652	− 5.659	− 31.216	− 23.248	− 2.290921	Asp F:343, phe F:346, gln F:349, asp F:340, leu F:339, lys F:338, gln E:260, pro E:258, gln F:306, phe F:307	
5.	2,3-dihydroxypropyl acetate	33510	− 3.74	− 30.282	− 25.124	− 1.455378	Lys F:338, leu F:339, asp F:340, asp F:343, pro E: 258, arg E:259, gln E:260, gln F:349, phe F:346, gln F:306, phe F: 307	
6.	Heptanoic acid, 6-oxo-	98810	− 3.109	− 28.494	− 20.932	3.398751	Lys F:338, leu F:339, asp F:340, asp F:343, Gln F:349, gln F:306, phe F:307, gln E:260, arg E:259, pro E:258	
7.	1-*O*-hexyl-d-glucitol	537891	− 0.802	− 32.419	− 34.68	8.658291	Ile F:337, lys F:338, leu F:339, asp F:340, asp F:341, lys F:342, asp F:343, asn F:345, gln F:349, gln F:306, phe F:307, pro E: 258, arg E:259, gln E:260	
8.	5H-Imidazole-4-carboxylic acid, 5-amino-, ethyl ester	6424292	− 3.695	− 27.143	− 21.765	4.204737	Pro E:258, arg E:259, gln E:260, ile F:337, lys F:338, leu F:339, asp F:340, asp F:343, phe F:307, gln F:306, gln F:349	
9.	Ethyl 5-oxo-2-pyrrolidinecarboxylate	2724446	− 6.705	− 38.468	− 27.525	1.857732	Gln F:349, phe F:307, gln F:306, asp F:343, asp F:340, leu F:339, lys F:338, gln E:260, arg E:259, pro E:258	
10.	Ethyl .alpha.-d-glucopyranoside	11127487	− 4.729	− 35.528	− 29.706	12.597659	Lys F:338, leu F:339, asp F:340, asp F:343, gln F:349, asn F:345, pro E: 258, gln E:260, lys E:261, phe F:307, gln F:306	
11.	3-Deoxy-D-mannoic lactone	541561	− 5.573	− 40.45	− 29.837	10.740183	Ile F:337, lys F:338, leu F:339, asp F:340, asp F:343, gln F:349, pro E:258, gln E:260, phe F:307, gln F:306	
12.	7,9-Di-tert-butyl-1-oxaspiro(4,5)deca-6,9-diene-2,8-dione	545303	-4.069	− 35.21	− 27.836	22.094183	Gln F:349, lys F:338, leu F:339, asp F:340, asp F:343, gln F:306, phe F:307, gln E:260, arg E:259, proE:258	
13.	gamma-Tocopherol	92729	− 2.941	− 38.779	− 34.731	27.74797	Gln F:349, lys F:338, leu F:339, asp F:340, asp F:343, asn F:345, lys E:257, pro E:258, arg E:259, gln E:260, lys E:261, phe F:307, gln F:306, ala F:305, pro F:302	
14.	3.alpha.,12.beta.-Dihydroxy-bisnor-5,7-cholenic acid	620404	− 4.46	− 37.004	− 33.064	29.544385	Arg E:262, gln E:260, arg E:259, pro E:258, lys E:257, lys F:338, leu F:339, asp F:340, asp F:343, asn F:345, gln F:306, phe F:307, gln F:349	
15.	DL-.alpha.-tocopherol	2116	− 3.681	− 42.953	− 37.729	28.147971	Asp F:343, asp F:340, leu F:339, lys F:338, gln F:349, phe F:307, gln F:306, ala F:305, pro F:302, lys E:261, gln E:260, arg E:259, pro E:258, lys E:257	
16.	Hexadecanoic acid, tetradecyl ester	78294	− 1.613	− 36.38	− 36.235	14.338562	Gln F:349, phe F:307, gln F:306, ala F:305, pro F:302, lys E:257, pro E:258, arg E:259, gln E:260, lys E:261, arg E:262, lys F:338, leu F:339, asp F:340, lys F:342, asp F:343, pro F:344	
17.	Ergost-5-en-3-ol, (3.beta.,24R)	6428659	− 2.939	− 27.614	− 23.806	64.747173	Lys F:338, leu F:339, asp F:340, asp F:343, asn F:345, gln E:260, pro E:258, phe F:307, gln F:306, gln F:349, gln F:303, pro F:302	
18.	Stigmasta-5,22-dien-3-ol	53870683	− 2.142	− 26.107	− 22.857	38.341859	Ser E:327, trp E:330, lys F:338, leu F:339, asp F:340, asp F:343, gly E:275, phe E:274, arg E:262, gln E:260, arg E:259, pro E:258, lys E:257	
19.	Fucosterol	5281328	− 2.019	− 25.696	− 23.984	40.224534	Trp E:330, lys F:338, asp F:340, asp F:343, arg E:262, gln E:260, arg E:259, pro E:258, lys E:257, trp E:330, gly E:275, phe E:274, ala E:273, gln E:272	
20.	Hydroxychloroquine	3652	− 3.302	− 34.237	− 30.378	41.642764	Pro E:258, arg E:259, gln E:260, phe F:307, gln F:349, ile F:337, lys F:338, leu F:339, asp F:340, lys F:342, asp F:343, pro F:344	

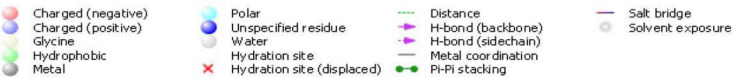

**Figure 3:**
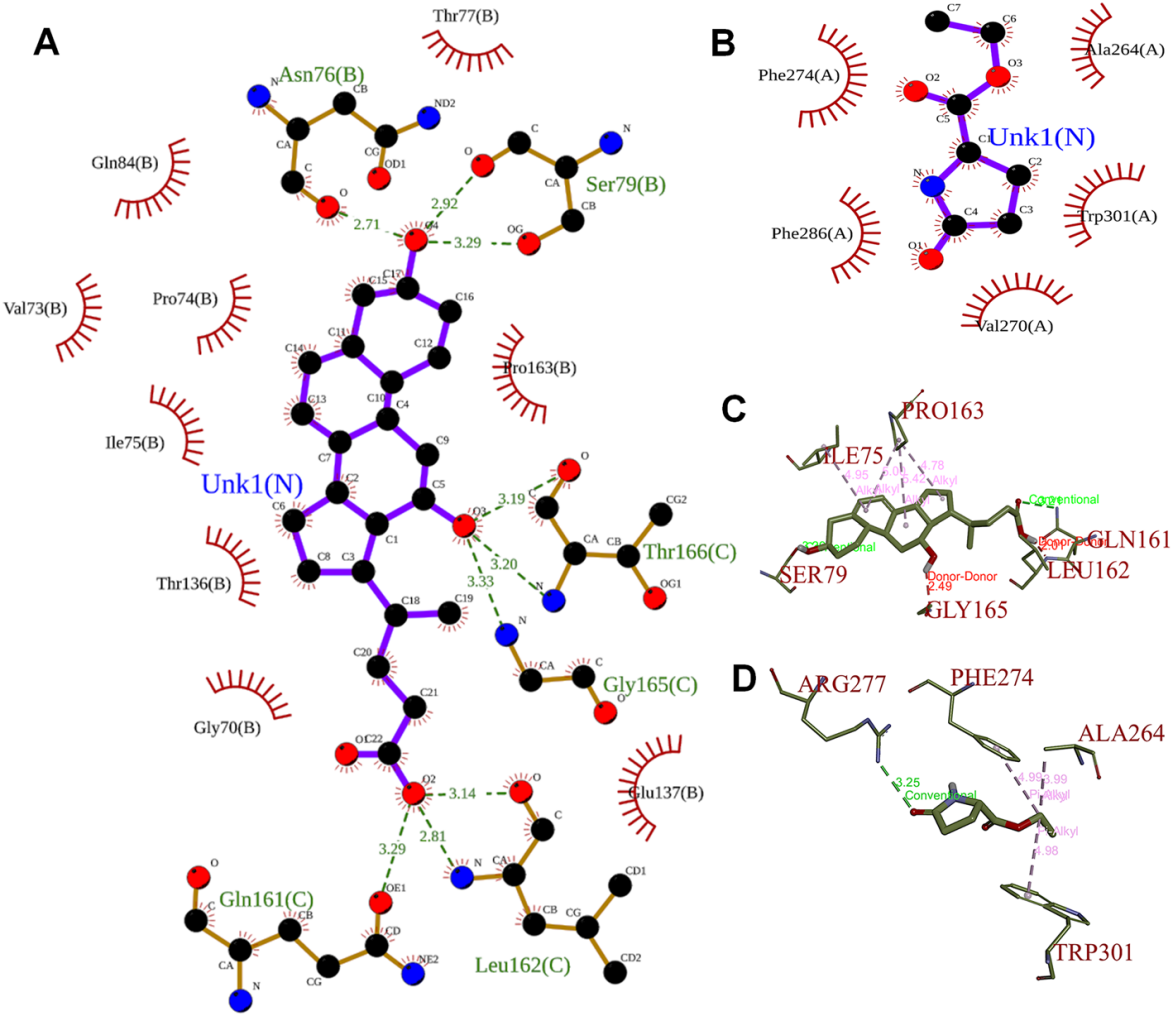
(**A**) Interaction between a best-docked complex of 3.alpha.,12.beta.-Dihydroxy-bisnor-5,7-cholenic acid of pomegranate seed and NRBD; PDB ID: 6M3M of SARS-CoV-2 using Ligplot. Only three residues (Leu162, H-bond = 2.81 Å, Gly 165, H-bond = 3.33 Å and Thr166, H-bond = 3.20 Å) was engaged in hydrogen bond formation between the N-group of protein and the O-group of ligands. (**B**) Interaction between the best-docked complex of ethyl 5-oxo-2-pyrrolidinecarboxylate and CTD; PDB ID: 6WJI of SARS-CoV-2 using Ligplot. No residue of protein was involved in H-bond formation, but all amino acid residues are linked with ligand through hydrophobic interaction. The half-circle’s reddish-brown colour denotes the protein residues engaged in the hydrophobic interaction with the ligand. The hydrogen bond is depicted by green dotted lines, and its bond length is indicated by the value. (**C** and **D**) It represents 3-D diagram by PyRx software of interacting amino acids residues between 3.alpha.,12.beta.-Dihydroxy-bisnor-5,7-cholenic acid with SARS-CoV-2 NRBD protein and ethyl 5-oxo-2-pyrrolidinecarboxylate molecule with CTD domain of SARS-CoV-2, respectively.

In ligand–protein complex, they bind with each other generally through non-covalent interactions viz. electrostatic, van der Waals forces, π–π interaction, and hydrophobic interaction^33^. The hydrophobic interactions increase the significance of protein binding as molecules become more lipophilic. But as evidenced by the interactions of numerous polar and nonpolar phytocomponents with specific proteins, many hydrophilic molecules are also electrostatically linked with protein molecules through ionic interactions and/or hydrogen bonds (Tables 2 and 3). As represented in Tables 2 and 3, twenty-one and eighteen hits, respectively had a good glide score > − 1.0 kcal/mol because of electrostatic attraction and hydrogen bonds between the hydroxyl groups of the ligand and various amino acid residues. As per results of Tables 2 and 3, the top twenty hits of pomegranate seeds with NRBD and CTD nucleocapsid proteins could be used to develop antiviral drugs against SARS-CoV-2 pathogenesis. The 2-D interaction of the amino acids Gln84, Val73, Pro74, Ile75, Thr136, Gly70, Glu137, Pro163, Thr77, Glu161, Leu162, Gly165, Thr166, Ser79, and Asn76 of a best-docked complex between 3.alpha.,12.beta.-Dihydroxy-bisnor-5,7-cholenic acid and SARS-CoV-2 NRBD protein is shown by the LigPlot (Fig. 3A). The O-group of ligand molecule is connected through three H-bonds with bond length 2.81 Å, 3.33 Å and 3.20 Å with N-group of Leu162, Gly165 and Thr166 amino acid residues of protein molecule. While, amino acid residues Gln84, Val73, Pro74, Ile75, Thr136, Gly70, Glu137, Pro163, and Thr77 were linked to the ligand through hydrophobic contact. Figure 3B illustrates the interaction between the amino acid residues Phe274, Phe286, Val270, Trp301 and Ala264 of CTD domain of SARS-CoV-2 and ethyl 5-oxo-2-pyrrolidinecarboxylate molecule of pomegranate seed. In this complex, all amino acid residues are linked with ligand through hydrophobic interaction. Figure 3C and D illustrate the 3-D diagram of interacting amino acids residues between 3.alpha.,12.beta.-Dihydroxy-bisnor-5,7-cholenic acid with SARS-CoV-2 NRBD protein and ethyl 5-oxo-2-pyrrolidinecarboxylate molecule with CTD domain of SARS-CoV-2 through PyRx software.

Different groups of phytochemicals such as Pyranone, Cyclopentane, Fatty acid ketone, Aromatic heterocycle (alkaloid), Amine, Glucopyranoside, Lactone derivative, Ketone, Sterol, Steroid, Vitamin E, Fatty acid ester and β-sitosterol (phytosterol) and their derivatives were identified as active constituents in PSE (Table 1). These constituents were selected as antiviral agents for in silico binding interaction towards NRBD and CTD nucleocapsid proteins of the SARS-CoV-2 and interestingly, these components showed greater affinity towards their targeted protein. Pyrones or pyranones are a class of heterocyclic chemical compounds which display chemotherapeutic potentials especially antibacterial and anticancer activity^34^. Cyclopentane or C pentane is a highly flammable alicyclic hydrocarbon which derivatives have also been shown the potent activity against hepatitis C and dengue viruses^35^. Oleic acid Ketones are cellular energy-sustaining endogenous compounds that also have drug-like signalling properties that influence immune response, metabolism, and epigenetics^36^. Aromatic heterocyclic compounds are analogous to benzene which has been proven to be more effective for treating a number of infectious and life-threatening disorders. It demonstrates a broad range of biological effects, including antiviral, antibacterial, anti-diabetic, anti-cancer, anti-inflammatory, and antifungal properties^37^. Amines, derived from ammonia, is an organic compound containing nitrogen atoms with a lone pair of electrons. Various amino acid and peptide-based antiviral agents have been developed as protease inhibitors in clinical practice^38^. An earlier patent investigation found that administering compositions comprising vitamin E, tocopherol, or a tocopherol derivative orally, subcutaneously, intramuscularly, intravenously, or intraperitoneally suppressed viral and retroviral reproduction^39^. Phytosterols are phytosteroids, similar to cholesterol, that are used by plants as structural elements in their biological membranes. Previous research has demonstrated the antioxidant, antibacterial, and antifungal properties of phytosterol found in the bark of Norway spruce *Picea abies*^40^. *Punica granatum* seed is a good source of antioxidants because of its high polyphenolic and flavonoids and vitamin C content^41^. As a result, it possesses both therapeutic as well as preventive properties from disease progression. Thus, natural chemicals found in pomegranate seeds could be used as preventive and/or therapeutic candidates in the drug discovery process based on these earlier results and current virtual in silico analysis.

### Molecular dynamics simulation analysis of ligand–protein complex

**Figure 4:**
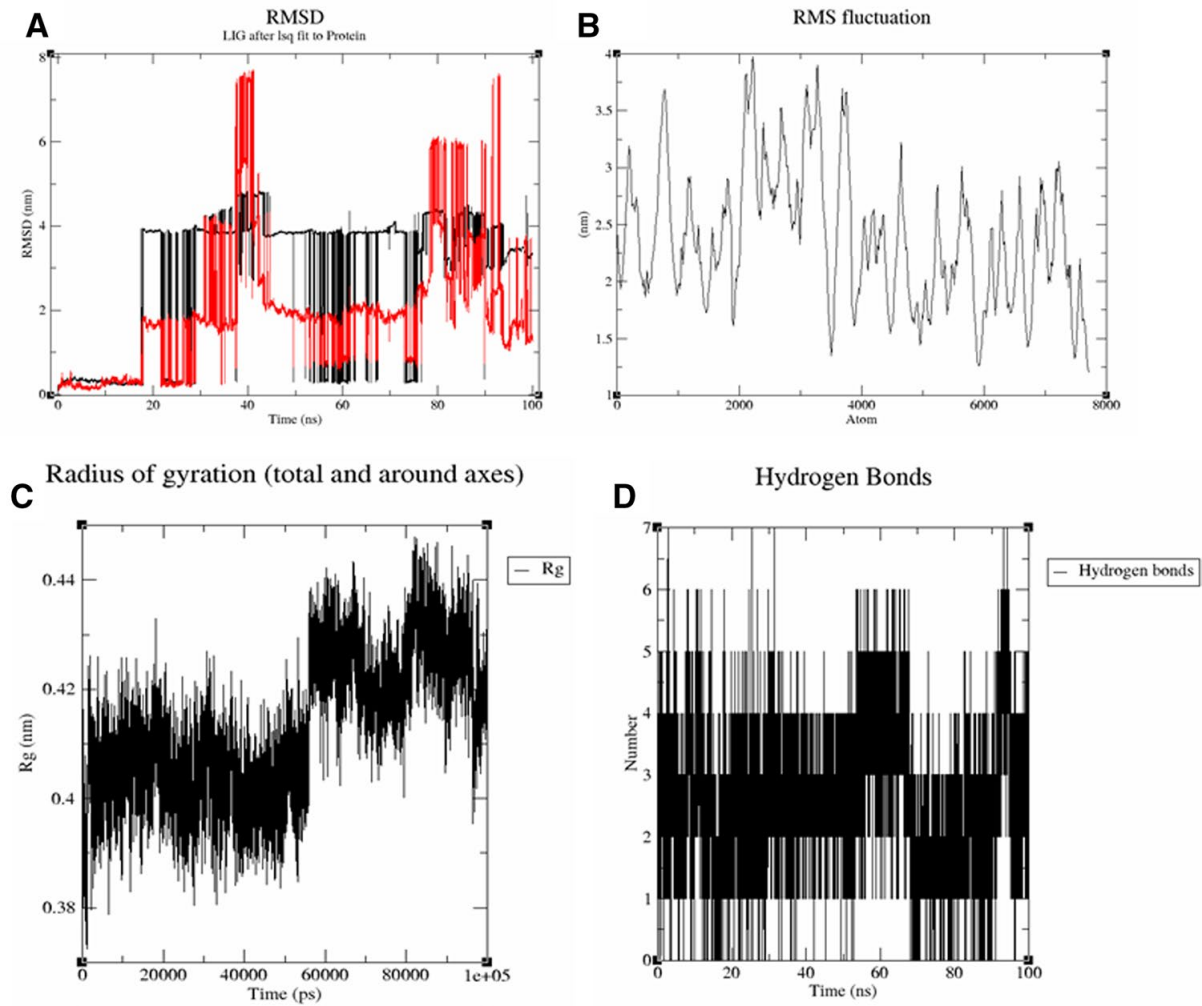
MD simulation trajectory of SARS-CoV-2 nucleocapsid protein NRBD for 100 ns MD simulation. (**A**) The RMSD of the SARS-CoV-2 nucleocapsid protein NRBD unbound (black) with alpha.,12.beta.-Dihydroxy-bisnor-5,7-cholenic acid (Red). (**B**) The RMSF value of SARS-CoV-2 nucleocapsid protein NRBD C-alpha atom. (**C**) Radius of gyration of alpha.,12.beta.-Dihydroxy-bisnor-5,7-cholenic acid. (**D**) Plot of Hydrogen Bond displaying number of bond per second between nucleocapsid protein NRBD and alpha.,12.beta.-Dihydroxy-bisnor-5,7-cholenic acid.

**Figure 5:**
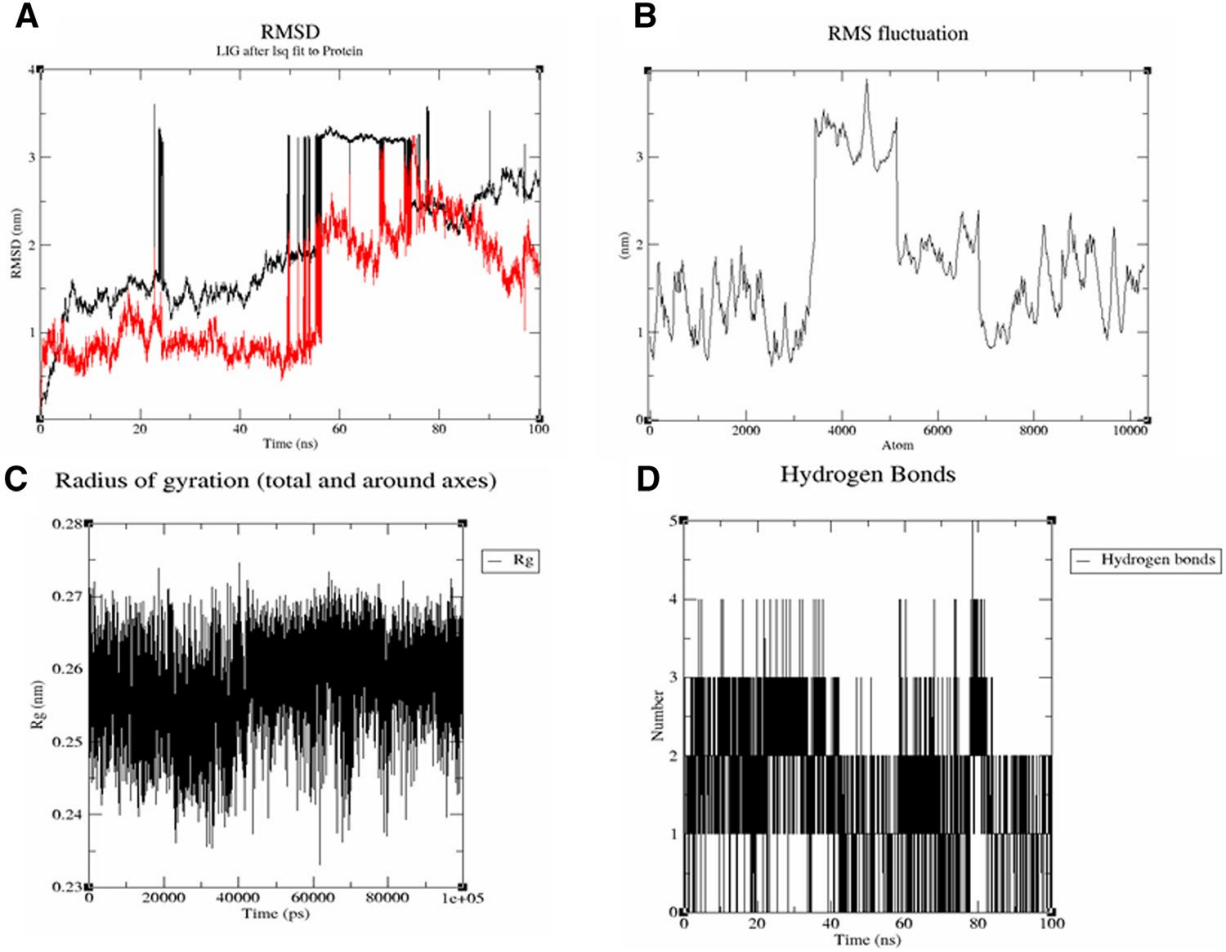
MD simulation trajectory of SARS-CoV-2 nucleocapsid CTD domain for 100 ns MD simulation. (**A**) The RMSD of the SARS-CoV-2 nucleocapsid CTD domain unbound (black) with ethyl 5-oxo-2-pyrrolidinecarboxylate (Red). (**B**) The RMSF value of SARS-CoV-2 nucleocapsid CTD C-alpha atom. (**C**) Radius of gyration of ethyl 5-oxo-2-pyrrolidinecarboxylate. (**D**) Plot of Hydrogen Bond displaying number of bond per second between nucleocapsid CTD domain and ethyl 5-oxo-2-pyrrolidinecarboxylate.

RMSD analysis was evaluated with c-alpha atom of protein and ligand fit on protein, demonstrating the stable interaction between protein and ligand. A good RMSD value depends on the protein and ligand having the best binding interactions and the lowest binding energy^42^. As shown in the Table 4 and Fig. 4A of RMSD trajectory, SARS-CoV-2 nucleocapsid protein NRBD C-alpha atoms were ranging between 0.117 and 4.879 nm, while ligand alpha.,12.beta.-Dihydroxy-bisnor-5,7-cholenic acid RMSD were ranged between 0.086 and 7.718 nm. Considering this data, the SARS-CoV-2 NRBD-alpha.,12.beta.-Dihydroxy-bisnor-5,7-cholenic acid complex was notably stable instead of rotational movement of alpha.,12.beta.-Dihydroxy-bisnor-5,7-cholenic acid in binding pocket of NRBD protein. RMSD analysis of SARS-CoV-2 CTD—ethyl 5-oxo-2-pyrrolidinecarboxylate complex was also found in stable configuration with protein C-alpha atom RMSD ranging between 0.09 and 3.607 nm with ligand RMSD between 0.132 and 3.251 nm (Table 4 and Fig. 5A). Moreover, RMSF C-alpha atom trajectory was analyzed for assessment of secondary structure fluctuation during the 100 ns MD simulation run. The RMSF values were ranging between 1.206 and 3.973 nm and 0.609–3.898 nm for C-alpha of SARS-CoV-2 nucleocapsid NRBD and SARS-CoV-2 nucleocapsid CTD proteins, respectively (Table 4, Figs. 4B and 5B). The radius of gyration (Rg) describes how atoms in a protein are distributed along its axis and how compact a molecule is at its center of mass. Rg for both ligands *i.e.* alpha.,12.beta.-Dihydroxy-bisnor-5,7-cholenic acid and ethyl 5-oxo-2-pyrrolidinecarboxylate were found below the 0.45 and 0.28 nm, respectively (Figs. 4C and 5C). The number of hydrogen bonds between protein and ligand were calculated for each frame of the simulation. In the SARS-CoV-2 nucleocapsid protein NRBD-alpha.,12.beta.-Dihydroxy-bisnor-5,7-cholenic acid complex the number of H-bonds were found maximum seven in number and most of the time frame was resonated between 2 and 4 bond (Fig. 4D). This resonated conformation made this complex notably stable throughout 100 ns MD simulation. In case of SARS-CoV-2 nucleocapsid CTD-ethyl 5-oxo-2-pyrrolidinecarboxylate complex, maximum five H-bonds were found in the complex while most of the time frame was resonating between 2 and 4 H-bonds (Fig. 5D).

**Table 4:** RMSD and RMSF properties of SARS-CoV-2 nucleocapsid NRBD protein and CTD domain with alpha.,12.beta.-Dihydroxy-bisnor-5,7-cholenic acid and ethyl 5-oxo-2-pyrrolidinecarboxylate, respectively.

S. No.	Properties	NRBD protein- alpha.,12.beta.-Dihydroxy-bisnor-5,7-cholenic acid complex (value in nm)	CTD domain—ethyl 5-oxo-2-pyrrolidinecarboxylate complex (value in nm)
1.	RMSD protein C-alpha	0.117–4.879	0.09–3.607
2.	RMSD lig-fit on protein	0.086–7.718	0.132–3.251
3.	RMSF protein C-alpha	1.206–3.973	0.609–3.898
4.	Radius of gyration	0.45	0.28

### Physicochemical properties and toxicity profile of phytocomponents

Further, the phytoconstituents were analyzed using an offline version of Data Warrior 5.2.1 software and an online chemoinformatic application called Molinspiration to check the physicochemical characteristics and drug-likeness of molecules. Applying Lipinski's rule of five, Table 5 demonstrates the physicochemical characteristics of pomegranate seed constituents. Lipinski should only have committed one violation for an oral active compound^43^. Interestingly, 15 pomegranate seed phytoconstituents exhibited no violation and only one violation was exhibited by remaining five constituents. Standard drugs, ivermectin displayed two of Lipinski’s violations, while hydroxychloroquine exhibited zero violation. The percentage of medications that are absorbed represents the oral absorption, or the number of drugs that enter the bloodstream through the portal vein from the gastrointestinal lumen^44^. The results of activity spectra for substances reveals that the drug availability in the gut lumen is more than > 50% (Table 5; Column 1). TPSA has the potential to serve as effective molecular descriptor in the investigation of drug transport qualities like blood–brain barrier penetration and intestinal absorption. It can also be used as a reliable predictor of the bioavailability of medicinal compounds to the cells. TPSA is the aggregate of polar atoms (oxygen, nitrogen) and their corresponding hydrogen atoms’ contributions to a molecule's surface area, which would be generally van der Waals^45^. Molecules which are unable to penetrate cell membranes often have polar surface areas > 160 2. It typically takes a PSA of a molecule 90 2 to pass across the BBB and subsequently act on receptors in the central nervous system. It’s interesting to note that all phytoconstituents, as shown in Table 5, had TPSA values lower than 160, indicating adequate intestinal wall absorption. The phytoconstituents of pomegranate seeds also adhered to the other characteristics of Lipinski’s rule of 5. The potential toxicity and drug-likeness of the phytocomponents found in pomegranate seeds are shown in Table 6. Except for cyclopentane, 1-acetyl-1,2-epoxy-Heptanoic acid, 6-oxo-1-*O*-hexyl-d-glucitol, fucosterol, and hydroxychloroquine, the results showed that all phytocomponents are safe to use and have no known toxicity in terms of mutagenic, tumorigenic, adverse effects on reproduction, and irritation. The benefits and cons of various components were only partially disclosed by the current computational analysis of pomegranate seed contents; nonetheless, the bulk of phytocomponents showed strong antiviral activity and drug-like qualities.

**Table 5:** PASS analysis and physicochemical properties of major active components of top twenty hits from pomegranate seed components and reference drugs.

S. No.	Phytoconstituents	% Absorption^a^ (> 50%)	Topological Polar Surface Area (Å)^2^ (TPSA)^b^ (< 160 Å)	MW (< 500)	c logP^c^ (< 5)	Heavy atom count (n atom)	Hydrogen Bond Donors (nOHNH) (≤ 5)	Hydrogen Bond Acceptors (nON) (≤ 10)	Number of Rotatable bonds (≤ 10)	Lipinski’s violation
1.	Imidazolidine-2,4,5-trione	80.43	82.80	114.06	− 1.56	8	2	5	0	0
2.	2,5-Dimethyl-4-hydroxy-3(2H)-furanone	92.94	46.53	128.13	0.55	9	1	3	0	0
3.	Cyclopentane, 1-acetyl-1,2-epoxy-	98.78	29.60	126.16	0.66	9	0	2	1	0
4.	2(3H)-Furanone, dihydro-4-hydroxy-	92.94	46.53	102.09	− 1.49	7	1	3	0	0
5.	2,3-dihydroxypropyl acetate	85.96	66.76	134.13	− 0.89	9	2	4	4	0
6.	Heptanoic acid, 6-oxo-	90.24	54.37	144.17	0.66	10	1	3	5	0
7.	1-*O*-hexyl-d-glucitol	70.92	110.37	266.33	− 0.03	18	5	6	11	1
8.	5H-Imidazole-4-carboxylic acid, 5-amino-, ethyl ester	75.25	97.80	126.12	− 1.41	9	5	5	1	0
9.	Ethyl 5-oxo-2-pyrrolidinecarboxylate	89.88	55.40	157.17	0.05	11	1	4	3	0
10.	Ethyl .alpha.-d-glucopyranoside	74.71	99.38	208.21	− 1.65	14	4	6	3	0
11.	3-Deoxy-d-mannoic lactone	78.98	86.99	162.14	− 1.63	11	3	5	1	0
12.	7,9-Di-tert-butyl-1-oxaspiro(4,5)deca-6,9-diene-2,8-dione	94.03	43.38	276.38	2.31	20	0	3	2	0
13.	gamma-Tocopherol	98.83	29.46	416.69	8.98	30	1	2	12	1
14.	3.alpha.,12.beta.-Dihydroxy-bisnor-5,7-cholenic acid	82.17	77.75	360.49	3.39	26	3	4	4	0
15.	Stigmast-5-en-3-ol, oleate	99.92	26.30	679.17	10.31	49	0	2	23	1
16.	DL-.alpha.-tocopherol	98.83	29.46	430.72	9.04	31	1	2	12	1
17.	Hexadecanoic acid, tetradecyl ester	99.92	26.30	452.81	9.93	32	0	2	28	1
18.	Ergost-5-en-3-ol, (3.beta.,24R)¬	102.02	20.23	400.69	8.30	29	1	1	5	0
19.	Stigmasta-5,22-dien-3-ol	102.02	20.23	412.70	7.87	30	1	1	5	0
20.	Fucosterol	102.02	20.23	412.70	7.69	30	1	1	5	0
21.	Hydroxychloroquine	92.30	48.4	335.9	3.08	23	2	4	9	0
22.	Ivermectin	50.32	170.09	875.11	5.41	62	3	14	8	2

Note:

^a^Percentage absorption was calculated as: % Absorption = 109−[0.345 × Topological Polar Surface Area].

^b^Topological polar surface area (defined as a sum of surfaces of polar atoms in a molecule).

^c^Logarithm of compound partition coefficient between n-octanol and water.

**Table 6:** Druglikeness and toxicity calculation of top twenty hits from pomegranate seed components as well as reference drugs.

S. No.	Compounds name	Drug-likeness	Mutant	Tumorigenic	Reproductive effective	Irritant
1.	Imidazolidine-2,4,5-trione	0.8165	N	N	N	N
2.	2,5-Dimethyl-4-hydroxy-3(2H)-furanone	− 0.8853	N	N	N	N
3.	Cyclopentane, 1-acetyl-1,2-epoxy-	− 2.297	N	L	H	N
4.	2(3H)-Furanone, dihydro-4-hydroxy-	− 0.2470	N	N	N	N
5.	2,3-dihydroxypropyl acetate	− 0.4205	N	N	L	N
6.	Heptanoic acid, 6-oxo-	− 13.79	N	N	N	H
7.	1-*O*-hexyl-d-glucitol	− 19.01	N	N	H	H
8.	5H-Imidazole-4-carboxylic acid, 5-amino-, ethyl ester	− 2.5139	N	N	N	N
9.	Ethyl 5-oxo-2-pyrrolidinecarboxylate	− 0.4895	N	N	N	N
10.	Ethyl.alpha.-d-glucopyranoside	− 10.47	N	N	N	N
11.	3-Deoxy-d-mannoic lactone	− 1.0679	N	N	N	N
12.	7,9-Di-tert-butyl-1-oxaspiro(4,5)deca-6,9-diene-2,8-dione	− 9.738	N	N	N	N
13.	gamma-Tocopherol	− 3.2757	N	N	N	N
14.	3.alpha.,12.beta.-Dihydroxy-bisnor-5,7-cholenic acid	0.34402	N	N	N	N
15.	Stigmast-5-en-3-ol, oleate	− 32.594	N	N	N	N
16.	dl-.alpha.-tocopherol	− 3.2757	N	N	N	N
17.	Hexadecanoic acid, tetradecyl ester	− 30.202	N	N	N	N
18.	Ergost-5-en-3-ol, (3.beta.,24R)	− 8.1908	N	N	N	N
19.	Stigmasta-5,22-dien-3-ol	1.2217	N	N	N	N
20.	Fucosterol	− 6.2842	N	N	N	H
21.	Hydroxychloroquine	5.7266	H	N	N	N
22.	Ivermectin	5.2314	N	N	N	N

N, no toxicity; L, low toxicity; H, high toxicity.

## Conclusions

GC–MS analysis of PSE showed thirty-two phytoconstituents of different groups such as pyranone, cyclopentane, fatty acid ketone, aromatic heterocycle (alkaloid), amine, glucopyranoside, lactone derivative, ketone, sterol, vitamin E, fatty acid ester and β-sitosterol (phytosterol) and their derivatives. These elements might be the underlying factor for antioxidant activity of PSE. The top twenty hits of PSE phytoconstituents displayed strong binding affinity with NRBD and CTD nucleocapsid protein of SARS-CoV-2. Remarkably, the majority of the phytoconstituents showed drug-like characteristics without any indication of toxicity. Based on this study, top twenty phytocomponents of pomegranate seed may be used as valued source for drug or nutraceutical development. More specifically based on Docking score and MD simulation analyzes, top two compounds, ethyl 5-oxo-2-pyrrolidinecarboxylate (a class of alpha amino acids derivatives) and 3.alpha., 12.beta-dihydroxy-bisnor-5,7-cholenic acid (phytosteroid derivative), could be explored further for their antiviral activity. These findings, however, urge for more refinement and confirmation through preclinical research to combat SARS-CoV-2 pathogenesis.

### Supplementary Information

Supplementary Table S1.

Supplementary Table S2.

## Data Availability

The datasets used and/or analyzed during the current study available from the corresponding authors MAB and SS on reasonable request.
