# Supplementary material for: Phytocompound screening, antioxidant activity and molecular docking studies of pomegranate seed: a preventive approach for SARS-CoV-2 pathogenesis
Source: Sci Rep. 2023 Oct 10;13:17069. doi: 10.1038/s41598-023-43573-1 (PMC10564957; doi:10.1038/s41598-023-43573-1)
Supplement: Supplementary file 1 — Supplementary Table S1. [file 41598_2023_43573_MOESM1_ESM.docx]

**Table S1.** Docking score, Glide E model, Glide energy, Gibbs binding free energy, interacting amino acids and 2-D interaction diagram of the docked ligand-protein complex of PSE components as well as standard drug with crystal structure of N-terminal RNA binding domain (NRBD; PDB ID: 6M3M) of nucleocapsid protein from SARS-CoV-2 using glide SP module of Schrödinger Maestro Release 2020-2

| **S. No.** | **Ligands** | **PubChem CID** | **Docking score**  **(kcal/mol)** | **Glide E model**  **(kcal/mol)** | **Glide energy**  **(kcal/mol)** | **Gibbs binding**  **free energy**  **(kcal/mol)** | **Interacted amino acid** | **2-D structure of ligand-protein interaction** |
| --- | --- | --- | --- | --- | --- | --- | --- | --- |
|  | 4H-Pyran-4-one, 2,3-dihydro-3,5-dihydroxy-6-methyl­ | 119838 | - | - | - | - | - | - |
|  | 2-Butanone, 4-hydroxy-3-methyl­ | 18829 | - | - | - | - | - | - |
|  | 2-Furancarboxaldehyde, 5-(hydroxymethyl)­ | 237332 | - | - | - | - | - | - |
|  | Phenol, 2,4-bis(1,1-dimethylethyl)­ | 7311 | - | - | - | - | - | - |
|  | n-Hexadecanoic acid | 985 | 0.262 | -27.806 | -31.881 | 9.373 | Tyr D:113, Ile D:147, Phe D:54, Trp D:53, Asn D:76, Thr D:77, Asn D:78, Ser D:79, Ile D:158, Asn D:155, Asn B:127, Asn A:155, Asn A:154, Asn A:151, Arg A:150, Thr A:149, Trp A:53, Thr A:50, Asn A:49 | 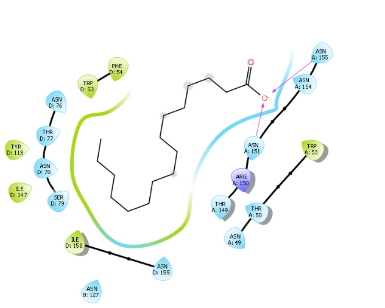 |
|  | Octadecanal | 12533 | 0.125 | -30.379 | -32.512 | 9.955 | Asn D:155, Ala D:156, Ala D:157, Ile D:158, Val D:159, Gln D:161, Thr A:149, Gly A:148, Ile A:147, Hie A:146, Asp A:145, Ser D:79, Asn D:78, Asn D:76, Trp D:53, Thr A:50, Asn A:49, Asn B:127, Ala B:126, Gly A:125, Ile D:147, Hie D:146 | 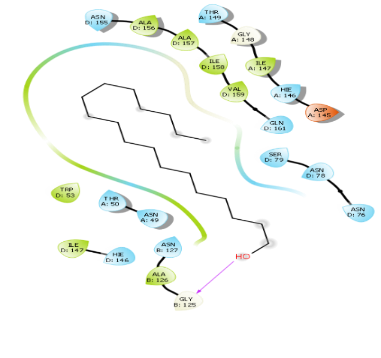 |
|  | Cholesta-4,6-dien-3-ol, (3.beta.)­ | 14795191 | - | - | - | - | - | - |
|  | Stigmast-5-en-3-ol, oleate | 20831071 | - | - | - | - | - | - |
|  | Stigmast-5-en-3-ol, (3.beta.)­ | 6432744 | - | - | - | - | - | - |
|  | Ivermectin | 6321424 | - | - | - | - | - | - |


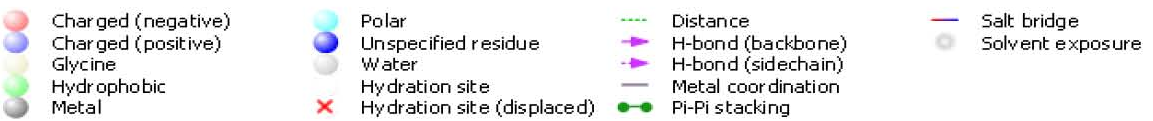
Note:
