# Supplementary material for: Phytocompound screening, antioxidant activity and molecular docking studies of pomegranate seed: a preventive approach for SARS-CoV-2 pathogenesis
Source: Sci Rep. 2023 Oct 10;13:17069. doi: 10.1038/s41598-023-43573-1 (PMC10564957; doi:10.1038/s41598-023-43573-1)

**Table S2.** Docking score, Glide E model, Glide energy, Gibbs binding free energy, interacting amino acids and 2-D interaction diagram of the docked ligand-protein complex of PSE phytocomponents as well as standard drug with C-terminal Dimerization Domain (CTD; PDB ID: 6WJI) of nucleocapsid protein from SARS-CoV-2 using glide SP module of Schrödinger Maestro Release 2020-2

| **S. No.** | **Ligands** | **PubChem CID** | **Docking score**  **(kcal/mol)** | **Glide E model**  **(kcal/mol)** | **Glide energy**  **(kcal/mol)** | **Gibbs binding**  **free energy**  **(kcal/mol)** | **Interacted amino acid** | **2-D structure of ligand-protein interaction** |
| --- | --- | --- | --- | --- | --- | --- | --- | --- |
|  | 4H-Pyran-4-one, 2,3-dihydro-3,5-dihydroxy-6-methyl­ | 119838 | - | - | - | - | - | - |
|  | 2-Butanone, 4-hydroxy-3-methyl­ | 18829 | - | - | - | - | - | - |
|  | 2-Furancarboxaldehyde, 5-(hydroxymethyl)­ | 237332 | - | - | - | - | - | - |
|  | Phenol, 2,4-bis(1,1-dimethylethyl)­ | 7311 | - | - | - | - | - | - |
|  | Tetradecanoic acid | 11005 | 1.143 | -18.242 | -25.943 | 7.845974 | Lys E:257, pro E:258, arg E:259, gln E:260, lys E:261, lys F:338, leu F:339, asp F:340, lys F:342, asp F:343, phe F:307, gln F:306, gln F:349 | 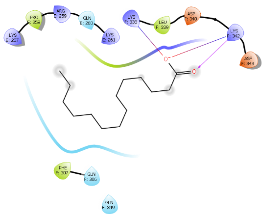 |
|  | n-Hexadecanoic acid | 985 | 0.94 | -22.826 | -29.963 | 9.373402 | Gln F:306, phe F:307, gln F:349, asp F:343, lys F:342, asp F:340, leu F:339, lys F:338, lys E:257, pro E:258, arg E:259, gln E:260, lys E:261 | 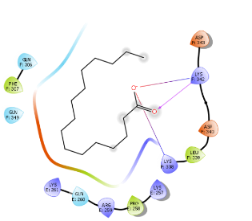 |
|  | Hexadecanoic acid, ethyl ester | 12366 | 1.84 | -19.619 | -27.138 | 10.3097 | Lys E:261, gln E:260, pro E:258, ile F:337, lys F:338, leu F:339, asp F:240, asp F:343, gln F:349, phe F:307, gln F:306, ala F:305, pro F:302, trp F:301, thr F:296, ser E:312, ala E:311 | 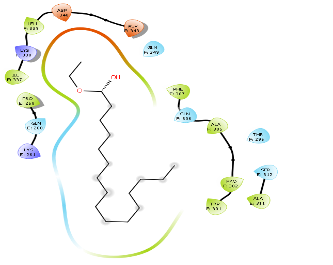 |
|  | Oleic acid | 445639 | 1.07 | -20.459 | -29.219 | 2.677625 | Phe F:307, gln F:306, asp F:343, asp F:340, leu F:339, lys F:338, gln F:349, trp E:330, pro E:258, arg E:259, gln E:260, lys E:261, arg E:262 | 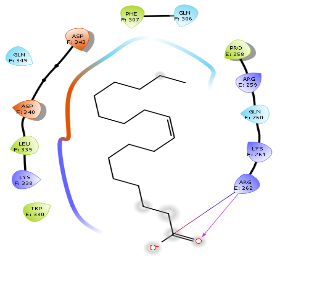 |
|  | Octadecanoic acid | 5281 | 2.304 | -20.731 | -27.463 | 4.76791 | Gln F:349, asp F:343, asp F:340, leu F:339, lys F:338, pro E:258, gln E:260, lys E:261, phe F:307, gln F:306, ala F:305, pro F:302, trp F:301 | 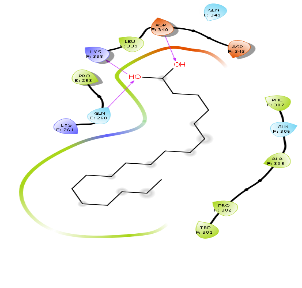 |
|  | Octadecanal | 12533 | 2.184 | -21.358 | -27.743 | 9.954903 | Gln F:349, phe F:346, asp F:343, asp F:340, leu F:339, pro E:258, gln E:260, lys E:261, thr F:296, phe F:307, gln F:306, ala F:305, pro F:302, trp F:301 | 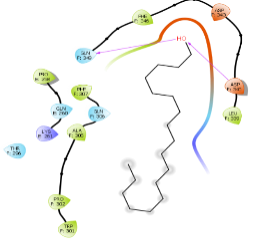 |
|  | Cholesta-4,6-dien-3-ol, (3.beta.)­ | 14795191 | - | - | - | - | - | - |
|  | Stigmast-5-en-3-ol, oleate | 20831071 | - | - | - | - | - | - |
|  | Stigmast-5-en-3-ol, (3.beta.)­ | 6432744 | - | - | - | - | - | - |
|  | Ivermectin | 6321424 | - | - | - | - | - | - |


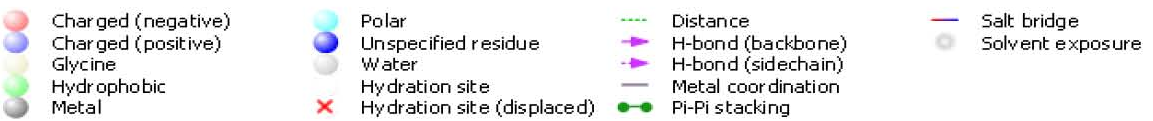

Supplement: Supplementary file 2 — Supplementary Table S2. [file 41598_2023_43573_MOESM2_ESM.docx]
